# Poly‐GP in cerebrospinal fluid links *C9orf72*‐associated dipeptide repeat expression to the asymptomatic phase of ALS/FTD

**DOI:** 10.15252/emmm.201607486

**Published:** 2017-04-13

**Authors:** Carina Lehmer, Patrick Oeckl, Jochen H Weishaupt, Alexander E Volk, Janine Diehl‐Schmid, Matthias L Schroeter, Martin Lauer, Johannes Kornhuber, Johannes Levin, Klaus Fassbender, Bernhard Landwehrmeyer, German Consortium for Frontotemporal Lobar Degeneration, Martin H Schludi, Thomas Arzberger, Elisabeth Kremmer, Andrew Flatley, Regina Feederle, Petra Steinacker, Patrick Weydt, Albert C Ludolph, Dieter Edbauer, Markus Otto, Adrian Danek, Emily Feneberg, Sarah Anderl‐Straub, Christine von Arnim, Holger Jahn, Anja Schneider, Manuel Maler, Maryna Polyakova, Lina Riedl, Jens Wiltfang, Georg Ziegler

**Affiliations:** ^1^ German Center for Neurodegenerative Diseases (DZNE) and Munich Cluster for System Neurology (SyNergy)MunichGermany; ^2^ Department of NeurologyUlm University HospitalUlmGermany; ^3^ Institute of Human GeneticsUniversity Medical Centre Hamburg‐EppendorfHamburgGermany; ^4^ Department of Psychiatry and PsychotherapyTechnical University of MunichMünchenGermany; ^5^ Clinic for Cognitive NeurologyUniversity Clinic LeipzigLeipzigGermany; ^6^ Max Planck Institute for Human Cognitive and Brain SciencesLeipzigGermany; ^7^ Department of Psychiatry, Psychosomatics and PsychotherapyUniversity Hospital of WürzburgWürzburgGermany; ^8^ Department of Psychiatry and PsychotherapyFriedrich‐Alexander‐University of Erlangen‐NurembergErlangenGermany; ^9^ Department of NeurologyLudwig‐Maximilians‐University Universität MünchenMunichGermany; ^10^ Department of NeurologySaarland UniversityHomburgGermany; ^11^ Center for Neuropathology and Prion ResearchLudwig‐Maximilians‐University MunichMunichGermany; ^12^ Department of Psychiatry and PsychotherapyLudwig‐Maximilians‐University MunichMunichGermany; ^13^ Institute of Molecular ImmunologyHelmholtz Zentrum MünchenGerman Research Center for Environmental Health (GmbH)MunichGermany; ^14^ Monoclonal Antibody Core Facility and Research GroupInstitute for Diabetes and ObesityHelmholtz Zentrum MünchenGerman Research Center for Environmental Health (GmbH)MunichGermany; ^15^ Department of Neurodegenerative Diseases and GerontopsychiatryBonn University HospitalBonnGermany

**Keywords:** amyotrophic lateral sclerosis, biomarker, *C9orf72*, cerebrospinal fluid, frontotemporal dementia, Biomarkers & Diagnostic Imaging, Genetics, Gene Therapy & Genetic Disease, Neuroscience

## Abstract

The *C9orf72* GGGGCC repeat expansion is a major cause of amyotrophic lateral sclerosis and frontotemporal dementia (c9ALS/FTD). Non‐conventional repeat translation results in five dipeptide repeat proteins (DPRs), but their clinical utility, overall significance, and temporal course in the pathogenesis of c9ALS/FTD are unclear, although animal models support a gain‐of‐function mechanism. Here, we established a poly‐GP immunoassay from cerebrospinal fluid (CSF) to identify and characterize *C9orf72* patients. Significant poly‐GP levels were already detectable in asymptomatic *C9orf72* mutation carriers compared to healthy controls and patients with other neurodegenerative diseases. The poly‐GP levels in asymptomatic carriers were similar to symptomatic c9ALS/FTD cases. Poly‐GP levels were not correlated with disease onset, clinical scores, and CSF levels of neurofilaments as a marker for axonal damage. Poly‐GP determination in CSF revealed a *C9orf72* mutation carrier in our cohort and may thus be used as a diagnostic marker in addition to genetic testing to screen patients. Presymptomatic expression of poly‐GP and likely other DPR species may contribute to disease onset and thus represents an alluring therapeutic target.

## Introduction

Amyotrophic lateral sclerosis (ALS) and frontotemporal dementia (FTD) are neurodegenerative diseases with similar neuropathological features and overlapping clinical symptoms and pathomechanisms (Ling *et al*, 2013). To date, a genetic cause can be identified in around two‐thirds of familial and 10% of sporadic ALS (Renton *et al*, 2014). Similarly, a genetic cause is described in about 25% of familial and 10% of sporadic FTD (Belzil *et al*, 2016). The most frequent genetic cause of ALS, FTD, or a combination of both is a large GGGGCC repeat expansion in the *C9orf72* gene (c9ALS/FTD). Three non‐mutually exclusive mechanisms are discussed to mediate the effects of the hexanucleotide expansion. The C9orf72 protein has been linked to autophagy and its expression is reduced in ALS/FTD patients (Sellier *et al*, 2016). While *C9orf72* knockout mice show no neurodegeneration, repeat expressing mice develop neuron loss and TDP‐43 pathology depending on the expression levels (Hayes & Rothstein, 2016; Jiang *et al*, 2016; O'Rourke *et al*, 2016). Formation of repeat RNA foci in the nucleus and the accompanying sequestration of RNA‐binding proteins are thought to alter RNA processing (DeJesus‐Hernandez *et al*, 2011). The expanded repeat is translated into aggregating dipeptide repeat proteins (DPRs) by a non‐conventional mechanism termed repeat‐associated non‐ATG (RAN) translation (Ash *et al*, 2013; Mori *et al*, 2013b; Zu *et al*, 2013), which was first discovered for expanded CAG repeats (Zu *et al*, 2011).

Five DPR species result from the translation from sense (poly‐GA, poly‐GP, poly‐GR) and antisense RNA (poly‐PA, poly‐PR, and further poly‐GP) in all reading frames (Gendron *et al*, 2013; Mori *et al*, 2013a; Zu *et al*, 2013). DPRs accumulate in p62‐positive but TDP‐43‐negative neuronal inclusions in the brain, a pathognomonic feature of c9ALS/FTD (Al‐Sarraj *et al*, 2011; Mori *et al*, 2013b). *In vitro* and *in vivo* studies showed toxicity of the different DPR species by inhibition of gene expression, nucleocytoplasmic transport, and the ubiquitin‐proteasome system (May *et al*, 2014; Zhang *et al*, 2014; Jovicic *et al*, 2015). Poly‐GA is the most abundant DPR species in the brain whereas overexpression of the arginine‐containing species (poly‐GR/‐PR) causes the most severe toxicity in cellular and fly models (Mizielinska *et al*, 2014; Schludi *et al*, 2015).

However, in end‐stage brains DPR pathology does not correlate with the degree of neurodegeneration, which challenges the concept of DPRs as the driving force of acute neurodegeneration as overly simplistic (Mackenzie *et al*, 2013), although mouse models strongly support a gain‐of‐function mechanism (Chew *et al*, 2015; Jiang *et al*, 2016; Liu *et al*, 2016). However, post‐mortem studies cannot provide conclusions on the temporal sequence of events (DPR/TDP‐43 deposition and neurodegeneration). Neuropathological reports from rare cases suggest that DPRs accumulate in the brain prior to TDP‐43 pathology early in disease or even prior its onset (Baborie *et al*, 2014; Proudfoot *et al*, 2014; Vatsavayai *et al*, 2016). Thus, the study of DPR expression in the asymptomatic phase of *C9orf72* mutation carriers is essential to clarify the role of DPRs in the pathogenesis of c9ALS/FTD. So far only poly‐GP has been detected in cerebrospinal fluid (CSF) in a small case series of symptomatic c9ALS patients (Su *et al*, 2014). It is unclear how accurately CSF levels of poly‐GP reflect the overall DPR load, but it is currently the only way to analyze RAN translation in living patients.

Therefore, we performed a cross‐sectional study of CSF samples of patients in different stages of the disease, even before onset of either dementia or motor symptoms to elucidate the temporal course of poly‐GP expression in c9ALS/FTD pathogenesis. In addition, we correlated poly‐GP levels with clinical scores (ALSFRS‐R, FTLD‐CDR), markers of neurodegeneration/axonal damage (neurofilament light chain, NfL; phosphorylated neurofilament heavy chain, pNfH), age at disease onset, disease duration at CSF collection, and estimated repeat length to assess the interaction between DPR load and disease severity.

## Results

### Monoclonal antibodies specifically detect poly‐GP

**Figure 1:**
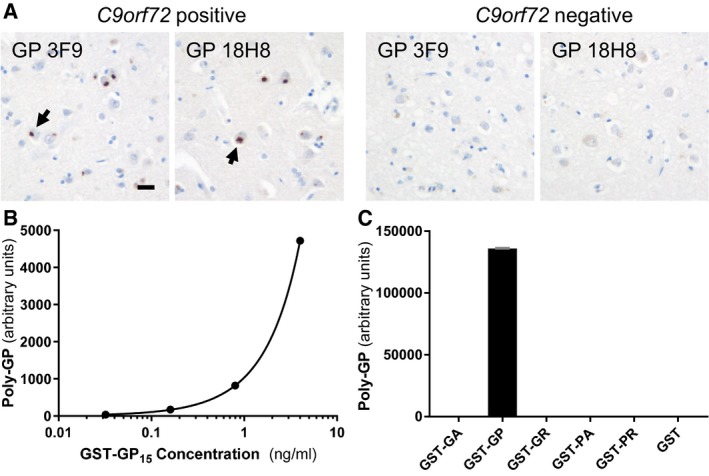
Validation of a novel poly‐GP‐specific immunoassay AImmunohistochemistry of frontal cortex from ALS/FTD cases with or without *C9orf72* repeat expansion using poly‐GP antibodies 18H8 and 3F9. Both antibodies detect neuronal cytoplasmic inclusions specifically in the *C9orf72* case (arrows). Hybridoma supernatants were used at 1:250 dilution as described previously (Schludi *et al*, 2015). Scale bar 20 μm.B, CPoly‐GP sandwich immunoassay with anti‐GP antibodies 18H8 and 3F9 detects purified GST‐GP_15_ below 0.03 ng/ml (B), but no other 15‐mer DPRs fused to GST at 1 μg/ml. Data are shown as mean ± SD (*n* = 2) (C). A four‐parameter logistic curve was used to fit the dose–response using Prism 7.01 software.

**Figure EV1:**
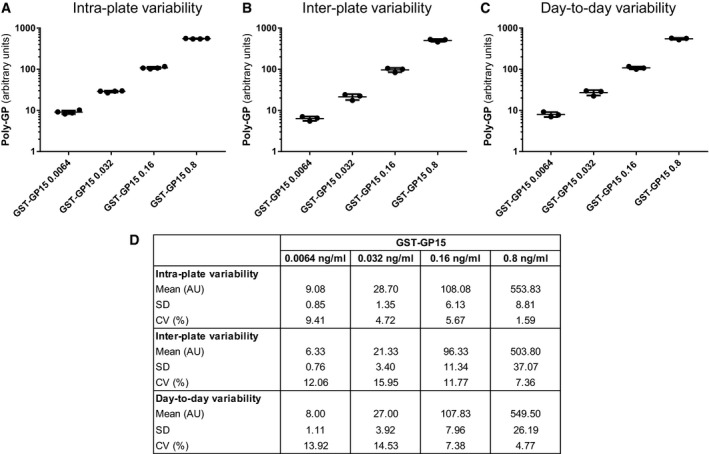
The poly‐GP immunoassay is reproducible A–DPoly‐GP sandwich immunoassay with anti‐GP antibodies 18H8 and 3F9 was used to analyze the GST‐GP_15_ standard at four concentrations. Background‐corrected absolute values, mean, and standard deviation (SD) for *n* = 4 GST‐GP_15_ intra‐plate replicates (A), *n* = 3 inter‐plate replicates (B), and *n* = 3 day‐to‐day replicates (C). Mean, SD, and the coefficient of variance (CV) for all conditions are listed in (D).

To develop an anti‐GP sandwich immunoassay with optimal sensitivity, we rescreened all our monoclonal anti‐GP clones from rat for affinity as a capture antibody (Schludi *et al*, 2015). As expected, the two best anti‐GP clones 18H8 and 3F9 specifically detected neuronal cytoplasmic poly‐GP inclusions by immunohistochemistry in a c9ALS/FTD patient, but not in a *C9orf72*‐negative ALS/FTD case (Fig 1A). An optimized immunoassay using these antibodies reliably detected GST‐GP_15_ down to a concentration of 0.03 ng/ml (Figs 1B and EV1). No cross‐reactivity with other GST‐DPR fusion proteins was observed even at 1 μg/ml (Fig 1C). Due to the different number of epitopes in the GST‐GP_15_ and endogenous poly‐GP from patients with variable repeat length, we present only background‐corrected raw values of CSF samples. To confirm assay stability, we repeatedly measured the concentration of four recombinant GST‐GP_15_ calibration samples ranging from 0.0064 to 0.8 ng/ml (Fig EV1). The coefficient of variance was between 1.59 and 9.41% for intra‐plate replicates, between 7.36 and 15.95% for inter‐plates replicates, and between 4.77 and 14.53% for day‐to‐day replicates, suggesting the assay is sufficiently accurate for diagnostic use.

### Poly‐GP is detectable in the CSF of asymptomatic and symptomatic c9ALS/FTD

**Figure 2:**
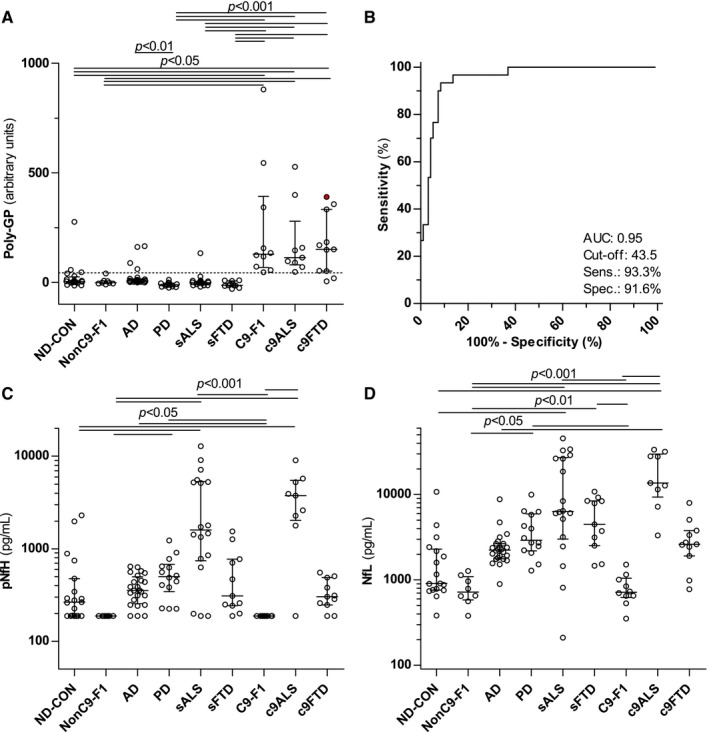
Poly‐GP expression is increased in CSF of asymptomatic and symptomatic *C9orf72* mutation carriers APoly‐GP was measured using immunoassay in an age‐matched control population without signs of a neurodegenerative disease (ND‐CON, *n* = 18–20), *C9orf72*‐negative offspring of *C9orf72* mutation carriers (NonC9‐F1, *n* = 8) in patients with other neurodegenerative diseases, that is, Alzheimer's (AD, *n* = 24) and Parkinson's disease (PD, *n* = 14), sporadic ALS (sALS, *n* = 18) and FTD (sFTD, *n* = 11) patients, and asymptomatic (C9‐F1, *n* = 10) and symptomatic *C9orf72* mutation carriers with ALS (c9ALS, *n* = 9) and FTD (c9FTD, *n* = 11). The c9FTD patient indicated by the filled, red circle was initially seen under the differential diagnosis of AD, but after poly‐GP measurement followed by *C9orf72* genotyping reclassified as c9FTD.BReceiver operating characteristic (ROC) curve analysis of poly‐GP levels for the discrimination of *C9orf72* mutation carriers vs. non‐carriers. The cutoff (43.5) was calculated using the Youden index and is shown as a dotted line in (A). AUC, area under the curve; Sens, sensitivity; Spec, specificity.C, D(C) Phosphorylated neurofilament heavy chain (pNfH) and (D) neurofilament light chain (NfL) were measured using an established ELISA.Data information: Groups were compared by Kruskal–Wallis test and Dunn's *post hoc* test. Bars and whiskers are median and interquartile range, and circles are individual values. Exact *P*‐values poly‐GP (A): ND‐CON vs. c9FTD: *P* = 0.0477; PD vs. AD: *P* = 0.0053; ND‐CON vs. c9ALS: *P* = 0.0483; ND‐CON vs. C9‐F1: *P* = 0.0236; NonC9‐F1 vs. c9FTD: *P* = 0.0365; NonC9‐F1 vs. c9ALS: *P* = 0.0334; NonC9‐F1 vs. C9‐F1: *P* = 0.0194; sALS vs. c9FTD: *P* = 0.0006; sALS vs. c9ALS: *P* = 0.0007; sALS vs. C9‐F1: *P* = 0.0003; sFTD vs. c9FTD, sFTD vs. c9ALS, sFTD vs. C9‐F1, PD vs. c9FTD, PD vs. c9ALS, and PD vs. C9‐F1: *P* < 0.0001. Exact *P*‐values pNfH (C): PD vs. C9‐F1: *P* = 0.0121; PD vs. NonC9‐F1: *P* = 0.0261; sALS vs. ND‐CON: *P* = 0.0103; C9‐F1 vs. AD: *P* = 0.0334; ND‐CON vs. c9ALS: *P* = 0.0142; NonC9‐F1 vs. c9ALS, C9‐F1 vs. c9ALS, sALS vs. C9‐F1, and sALS vs. NonC9‐F1: *P* < 0.0001. Exact *P*‐values NfL (D): sFTD vs. C9‐F1: *P* = 0.0013; sFTD vs. NonC9‐F1: *P* = 0.0038; PD vs. C9‐F1: *P* = 0.0122; PD vs. NonC9‐F1: *P* = 0.0245; c9ALS vs. AD: *P* = 0.0107; sALS vs. ND‐CON: *P* = 0.0017; sALS vs. NonC9‐F1: *P* = 0.0001; sALS vs. C9‐F1, ND‐CON vs. c9ALS, NonC9‐F1 vs. c9ALS, and C9‐F1 vs. c9ALS: *P* < 0.0001.

Poly‐GP levels were measured in CSF in a group of 125 clinically well‐characterized patients and controls from the German FTLD Consortium. The demographic characteristics of the participants are listed in Table 1. The sample includes 30 subjects with evidence of a repeat *C9orf72* expansion (C9‐F1, *n* = 10; c9ALS, *n* = 9; c9FTD, *n* = 11) in the peripheral blood. The median response in the poly‐GP immunoassay in the CSF of all 30 *C9orf72* patients was > 35‐fold higher than in ND‐CON and NonC9‐F1 controls (median 140.3, interquartile range 66.5 to 335.3 vs. median 4.0, interquartile range −1.25 to 24.9), which indicates a specific response (Fig 2A). We performed receiver operating characteristic (ROC) curve analysis of all *C9orf72* mutation carriers vs. all other samples. The area under the curve (AUC) was 0.95 (95% CI: 0.92–0.99) (Fig 2B) and at a cutoff of 43.5 the sensitivity was 93.3% (95% CI: 77.9–99.2%) and the specificity was 91.6% (95% CI: 84.1–96.3%).

**Table 1:** Patient characteristics

Characteristic	ND‐CON (*n* = 20)	NonC9‐F1 (*n* = 8)	AD (*n* = 24)	PD (*n* = 14)	sALS (*n* = 18)	sFTD (*n* = 11)	C9‐F1 (*n* = 10)	c9ALS (*n* = 9)	c9FTD (*n* = 11)
Age (years)^a^	63.5 (52.8 to 70.0)	42.3 (34.6 to 48.0)^b^	67.5 (56.6 to 70.2)	72.5 (67.0 to 77.0)	60.0 (52.0 to 67.5)	64.0 (53.0 to 68.0)	44.8 (39.4 to 51.2)^b^	65.1 (54.4 to 71.1)	56.3 (44.9 to 61.1)
Gender (F/M)	11/9	3/5	14/10	5/9	6/12	4/7	8/2	3/6	4/7
ALSFRS‐R^a^	n.a.	n.a.	n.a.	n.a.	41.0 (32.0 to 44.0)	n.a.	n.a.	39.0 (36.3 to 44.0)	n.a.
FTLD‐CDR^a^	n.a.	n.a.	n.a.	n.a.	n.a.	4.5 (1.0 to 5.5)	n.a.	n.a.	7.0 (3.8 to 11.8)
Disease duration at LP (months)^a^	n.a.	n.a.	n.a.	n.a.	14.5 (8.8 to 26.0)	21.0 (15.0 to 39.0)	n.a.	11.3 (4.8 to 29.9)	56.0 (23.4 to 163)^c^
Poly‐GP in CSF (arbitrary units)^a^	4.0 (−1.3 to 24.9)	−1.8 (−5.5 to 7.0)	6.0 (3.6 to 16.3)^d^	−10.5 (−18.9 to −3.6)	−1.3 (−9.6 to 5.3)	−13.5 (−16.0 to 7.0)	129 (68.0 to 393)^e^	113 (80.0 to 279)^f^	151 (51.5 to 333)^e^
NfL in CSF (pg/ml)^a^	909 (759 to 2,297)	720 (581 to 1,093)	2,232 (1,768 to 2,655)	2,911 (2,185 to 5,907)^g^	6,319 (3,000 to 27,013)^h^	4,455 (2,515 to 8,397)^i^	716 (620 to 1,043)	13,644 (9,313 to 29,818)^j^	2,614 (1,903 to 3,771)
pNfH in CSF (pg/ml)^a^	264 (188 to 474)	188 (188 to 188)	353 (254 to 495)^k^	499 (343 to 675)^l^	1,593 (790 to 5,325)^m^	309 (241 to 768)	188 (188 to 188)	3,740 (2,028 to 5,487)^n^	303 (246 to 485)

AD, Alzheimer's disease; ALS, amyotrophic lateral sclerosis; ALSFRS‐R, ALS Functional Rating Scale—revised; bvFTD, behavioral variant of frontotemporal dementia; C9‐F1, asymptomatic *C9orf72* mutation carriers; c9ALS, symptomatic ALS *C9orf72* mutation carriers; c9FTD, symptomatic bvFTD *C9orf72* mutation carriers; CSF, cerebrospinal fluid; F, female; FTLD‐CDR, Frontotemporal Lobar Degeneration‐specific Clinical Dementia Rating; LP, lumbar puncture; M, male; n.a., not available; ND‐CON, age‐matched control population without signs of a neurodegenerative disease; NfL, neurofilament light chain; NonC9‐F1, *C9orf72‐*negative offspring of a *C9orf72* mutation carrier; PD, Parkinson's disease; pNfH, phosphorylated neurofilament heavy chain; sALS, sporadic ALS; sFTD, sporadic bvFTD.

a Values are median and interquartile range.

b *P* < 0.05 vs. ND‐CON, *P* < 0.01 vs. AD, *P* < 0.001 vs. PD.

c *P* < 0.05 vs. sFTD.

d *P* < 0.01 vs. PD.

e *P* < 0.05 vs. ND‐CON, NonC9‐F1, *P* < 0.001 vs. sFTD, PD, sALS.

f *P* < 0.05 vs. ND‐CON, NonC9‐F1, *P* < 0.001 vs. sALS, sFTD, PD.

g *P* < 0.05 vs. NonC9‐F1, *P* < 0.01 vs. C9‐F1.

h *P* < 0.01 vs. ND‐CON, *P* < 0.001 vs. NonC9‐F1, C9‐F1.

i *P* < 0.001 vs. C9‐F1, NonC9‐F1.

j *P* < 0.01 vs. AD, *P* < 0.001 vs. ND‐CON, NonC9‐F1, C9‐F1.

k *P* < 0.05 vs. C9‐F1.

l *P* < 0.05 vs. NonC9‐F1, *P* < 0.01 vs. C9‐F1.

m *P* < 0.01 vs. ND‐CON, *P* < 0.001 vs. NonC9‐F1, C9‐F1.

n *P* < 0.05 vs. ND‐CON, *P* < 0.001 vs. NonC9‐F1, C9‐F1.

Of note, we detected poly‐GP signal in eight out of 95 from patients in the *C9orf72*‐negative groups. One patient who eventually received the clinical diagnosis of sporadic ALS and was initially seen under the differential diagnosis of hereditary spastic paraplegia (HSP) showed elevated poly‐GP levels in CSF. One ND‐CON patient with very high poly‐GP signal had undergone a lumbar puncture in order to exclude a chronic inflammatory process. This patient presented with dysaesthesia of the lower limbs, a small spinal lesion in MRI, but without oligoclonal bands or motor and frontal signs. The other patients included four patients with a clinical diagnosis of AD and two control patients with the clinical diagnosis of a vestibular neuritis and a polyneuritis. However, for the latter two patients, the poly‐GP levels were just above the calculated cutoff level. In these patients, there was no clinical sign for a neurodegenerative disease. The genetic *C9orf72* status of these patients was (re)analyzed except for the two control patients with vestibular neuritis and polyneuritis, where no DNA was available. We did not detect a *C9orf72* repeat expansion in peripheral blood, but cannot rule out a somatic mosaicism in the brain as autopsy samples were not available for a definitive diagnosis. In an additional poly‐GP‐positive AD case, genotyping indeed revealed a *C9orf72* mutation, which led to reclassification as c9FTD (Fig 2A, red dot).

Importantly, there was no significant difference in the poly‐GP levels of asymptomatic and symptomatic *C9orf72* mutation carriers and also not between c9ALS and c9FTD cases. In contrast, only c9ALS and sALS patients, but not C9‐F1 cases, showed increased CSF concentrations of pNfH and NfL (Fig 2C and D). Thus, poly‐GP in CSF is a biomarker for the identification of both symptomatic and asymptomatic *C9orf72* mutation carriers, while neurofilament levels in CSF are associated with the symptomatic but not the premanifest phase of the disease.

### Association of poly‐GP with other CSF biomarkers and clinical scales

**Figure 3:**
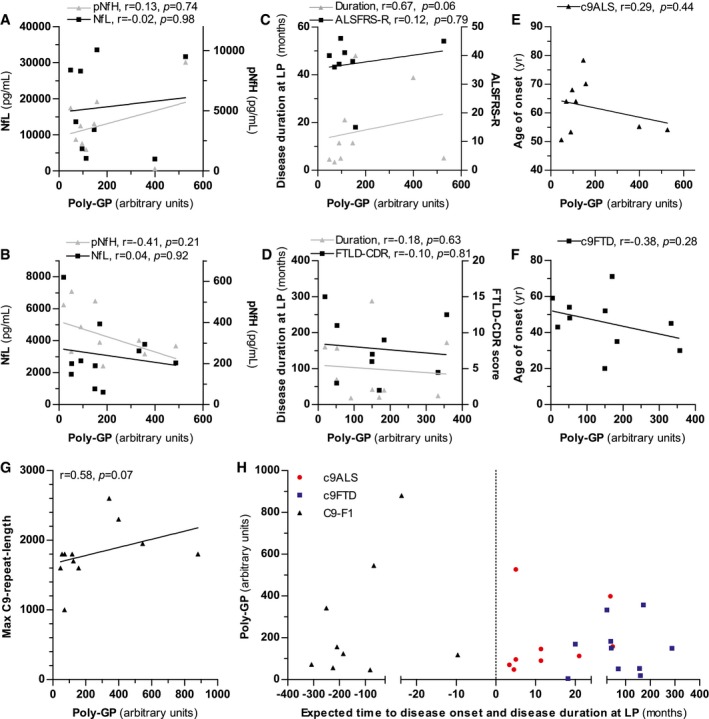
Poly‐GP expression in CSF correlates neither with markers of neurodegeneration nor with clinical disease severity A–FCorrelation analysis of poly‐GP levels in CSF of c9ALS (A, C, E) and c9FTD cases (B, D, F). Correlation with phosphorylated neurofilament heavy chain (pNfH) and neurofilament light chain (NfL) (A, B), with disease duration at lumbar puncture (LP) and the ALSFRS‐R or FTLD‐CDR score (C, D) and with age at disease onset (E, F).GCorrelation of poly‐GP levels in CSF with the largest repeat length estimated by Southern blotting.HAssociation of poly‐GP levels in CSF with disease duration at LP in c9ALS/FTD patients and with time to expected disease onset in C9‐F1 cases. Time to expected disease onset was calculated using parental age at disease onset.Data information: Correlation analysis was performed using Spearman’s rank correlation coefficient.

Next, we analyzed the correlation of poly‐GP levels in the CSF of c9ALS/FTD cases with different markers of neurodegeneration and disease severity (Fig 3). There was no significant correlation of poly‐GP levels with the axonal damage markers NfL (*r* = −0.02, *P* = 0.98 in c9ALS; *r* = 0.04, *P* = 0.92 in c9FTD) and pNfH (*r* = 0.13, *P* = 0.74 in c9ALS; *r* = −0.41, *P* = 0.21 in c9FTD) (Fig 3A and B). Furthermore, no significant correlation was observed with clinical scores (*r* = 0.12, *P* = 0.79 for ALSFRS‐R in c9ALS; *r* = −0.10, *P* = 0.81 for FTLD‐CDR in c9FTD), disease duration at the time of CSF collection (*r* = 0.67, *P* = 0.06 in c9ALS; *r* = −0.18, *P* = 0.63 in c9FTD) (Fig 3C and D), and age at disease onset (*r* = 0.29, *P* = 0.44 in c9ALS; *r* = −0.38, *P* = 0.28 in c9FTD) (Fig 3E and F). Current technologies allow only a rough estimate of the repeat length, because the expanded allele presents as a smear rather than a distinct band in Southern blots and somatic variability between blood and brain DNA is well described (Nordin *et al*, 2015). Given these limitations, no significant correlation of poly‐GP levels with the estimated repeat length from blood (available for 11 patients) was identified (*r* = 0.58, *P* = 0.07 for c9ALS and c9FTD combined) (Fig 3G).

Despite being a cross‐sectional study, we used a similar approach as that used in the GENFI study (Rohrer *et al*, 2015) in order to determine the changes of CSF poly‐GP throughout the evolution of the disease. That is, we used parental age of onset as a proxy to calculate the estimated years to disease onset. We did not find any association between the estimated years to disease onset and CSF poly‐GP (*r* = 0.28, *P* = 0.46) (Fig 3H). Thus, poly‐GP expression starts at least several years prior to clinical disease onset and remains unchanged in late stages.

## Discussion

Using a novel immunoassay, we measured poly‐GP in the CSF from *C9orf72* ALS and bvFTD cases and carefully selected control groups. Our main results were as follows: (i) Significant poly‐GP levels are detectable in the CSF of 93.3% of the *C9orf72* ALS and bvFTD cases but not in 91.6% of the control cases; (ii) rapid poly‐GP immunoassay is useful to detect individuals with a *C9orf72* expansion misdiagnosed with other diseases (e.g. AD); and (iii) poly‐GP levels are already increased in asymptomatic stages of the disease, suggesting DPRs may be most important for the early pathogenic events in *C9orf72* ALS/FTD rather than driving acute neurodegeneration in late‐stage patients.

### Poly‐GP immunoassay from CSF

Previously, poly‐GP had been detected in CSF of c9ALS cases by immunoassay using polyclonal antibodies (Su *et al*, 2014). Here, we developed an analogous immunoassay using two monoclonal anti‐GP antibodies. The monoclonal antibodies allow standardized analysis and are not vulnerable to limited antibody availability or batch‐to‐batch variation, which will be critical for the use as a therapeutic biomarker for repeat‐directed clinical trials (Jiang *et al*, 2016). The repeat expansion in *C9orf72* patients seems to vary mostly between 400 and > 5,000 (GGGGCC)_n_ repeats (Beck *et al*, 2013; Fratta *et al*, 2013) and is notoriously difficult to determine precisely (Akimoto *et al*, 2014). We present raw responses instead of absolute poly‐GP concentrations, because the repeat length affects epitope numbers and thus likely capture and detection of poly‐GP antigens in the immunoassay (compare Fig 3G). While low‐level release and intercellular transmission of all five DPR species have been reported in cell culture systems (Westergard *et al*, 2016), we have so far not been able to detect the other DPR species in patient CSF using a similar approach suggesting that these species might be released into the CSF at lower levels.

### Poly‐GP signal in apparently C9orf72‐negative cases

Using ROC analysis, we established a cutoff that allows sensitive and specific discrimination of most *C9orf72* cases from controls. Only two of our genetically verified c9ALS/FTD cases had low poly‐GP levels in CSF. In contrast, some of the non‐mutation carriers (one sALS, four AD, and one ND‐CON) showed strongly elevated poly‐GP signals. We offer three potential explanations. First, the repeat length in *C9orf72* patients is known to vary widely between different tissues (Nordin *et al*, 2015) and it is possible that the repeat length is normal in blood lymphocytes, but pathological in the central nervous system. Thus, somatic mosaicism could prevent detection of *bona fide C9orf72* cases using genotyping from peripheral blood. Emerging single‐cell genome data show an unexpected degree of mosaicism in health and disease (Forsberg *et al*, 2016). Second, other pathologically expanded repeats in the genome, for example, the intronic (GGCCTG)_n_ repeat expansion in the gene for the nucleolar protein *NOP56* causing spinocerebellar ataxia type 36 (SCA36), could result in poly‐GP expression (Kobayashi *et al*, 2011). Third, other CSF proteins with short poly‐GP stretches that are upregulated preferentially in a subgroup of AD patients may cross‐react in the immunoassay.

### High poly‐GP levels in presymptomatic C9orf72 carriers

Animal models support a predominant gain‐of‐function mechanism for *C9orf72* pathogenesis, but the role of DPR proteins in disease initiation and progression in human ALS and FTD patients remains unresolved. Here, we show that poly‐GP is already elevated in CSF of asymptomatic *C9orf72* mutation carriers ~14 years younger than the symptomatic group, suggesting that DPR expression is present in the earliest disease phase (compare Table 1). This is in agreement with the neuropathological detection of DPRs in presymptomatic *C9orf72* cases at young age (Baborie *et al*, 2014; Proudfoot *et al*, 2014; Vatsavayai *et al*, 2016). Interestingly, cross‐sectional data from the GENFI cohort show subtle brain volume loss and behavioral changes in *C9orf72* carriers already 20 years prior to the expected disease onset, while *MAPT* (microtubule‐associated protein tau) and *GRN* (granulin) mutation carriers show the first significant differences much closer to the disease onset (Rohrer *et al*, 2015). Presymptomatic DPR expression suggests that DPRs may be most critical for initially triggering the disease, while progression may largely depend on TDP‐43 pathology (Edbauer & Haass, 2016).

Moreover, poly‐GP levels are similar in c9ALS and c9FTD although disease duration is much shorter in ALS. Poly‐GP levels in CSF of c9ALS/FTD cases did not correlate with markers of neurodegeneration such as the axonal damage markers NfL and pNfH and with markers of disease severity (clinical scores, disease duration, and onset). This is consistent with neuropathological findings showing no spatial correlation of DPR pathology with neurodegeneration (Mackenzie *et al*, 2013; Schludi *et al*, 2015). It is unclear how CSF levels of poly‐GP correlate the amounts of poly‐GP and the other DPR species within the neuronal inclusions. Although the total DPR levels vary between patients, we are not aware of cases with vastly different ratios of the different DPR species (Mackenzie *et al*, 2015; Schludi *et al*, 2015).

Since poly‐GR/PR and poly‐GA are by far more toxic than poly‐GP in cellular and animal models (Mizielinska *et al*, 2014), it will be critical to determine their levels during disease progression to better address the role of DPRs in c9ALS/FTD pathogenesis.

In conclusion, poly‐GP determination in CSF may be used as an alternative or addition to genetic testing to identify *C9orf72* mutation carriers. Our data indicate that poly‐GP expression is already present in the presymptomatic phase of c9ALS/FTD, and thus, DPRs may predominantly contribute to triggering the disease rather than driving acute neurodegeneration in late‐stage patients. This has implications for developing drugs and designing clinical trials. A standardized monoclonal‐based anti‐GP immunoassay will be critical to determine whether antisense oligonucleotide treatment in patients reduces DPR expression in patients similar to the preclinical trials in mice (Su *et al*, 2014).

## Materials and Methods

### Patients

We investigated nine different patient groups: (i) symptomatic ALS *C9orf72* mutation carriers (c9ALS), (ii) symptomatic patients of the behavioral variant of FTD (bvFTD) *C9orf72* mutation carriers (c9FTD), (iii) asymptomatic *C9orf72* mutation carriers (C9‐F1), (iv) *C9orf72*‐negative offspring of a *C9orf72* mutation carrier (NonC9‐F1), (v) sporadic ALS patients (sALS), (vi) sporadic FTD patients (sFTD), two groups of other neurodegenerative diseases, namely (vii) Parkinson's disease (PD) and (viii) Alzheimer's disease (AD), and (ix) an age‐matched control population without clinical signs of a neurodegenerative disease (non‐neurodegenerative control, ND‐CON). Diagnosis was made according to standard criteria.

*C9orf72* ALS cases and NonC9‐F1 cases were recruited from the German Presymptomatic (GPS)‐ALS cohort (Weydt *et al*, 2016). AD and bvFTD patients (including *C9orf72* cases) were enrolled at different clinical centers coordinated by the German FTLD consortium (Erlangen, Leipzig, Munich, Ulm, Würzburg). All other patients were recruited at the Department of Neurology, Ulm University Hospital, Germany. Group size for the groups ND‐CON, PD, sALS, and sFTD was estimated by experience because no preliminary data were available. For the groups NonC9‐F1, AD, C9‐F1, c9ALS, and c9FTD, all samples available from the cohorts of the GPS‐ALS and FTLD consortium were used. All patients gave written informed consent. All procedures were in accordance with the WGA Declaration of Helsinki and the Department of Health and Human Services Belmont Report. The ethics committees of the participating centers approved the study (Otto *et al*, 2011).

All patients underwent neuropsychological testing using standard procedures. Disease severity in ALS patients was assessed using the ALS Functional Rating Scale—revised (ALSFRS‐R) and in bvFTD patients using the FTLD‐specific Clinical Dementia Rating (FTLD‐CDR) score. PCR‐based screening methods were used for the detection of *C9orf72* repeat expansion. If enough DNA was available, Southern blot analyses were conducted (Akimoto *et al*, 2014).

Cerebrospinal fluid was collected by lumbar puncture, centrifuged, and stored within 2 h at −80°C following standard operating procedures at all sites.

### Poly‐GP sandwich immunoassay from CSF

By immunizing Lou/c rats with synthetic GP_10_ peptides, the poly‐GP‐specific monoclonal antibodies 18H8 (IgG1/κ) and 3F9 (IgG2a/κ) were raised using previously described protocols (Mackenzie *et al*, 2013). These new monoclonal antibodies against poly‐GP had higher affinity than the previously described clone 7A5 (Schludi *et al*, 2015). An immunoassay was performed using the Meso Scale Discovery platform (MSD). Streptavidin plates (MSD Gold 96‐well Streptavidin) were coated with biotinylated 18H8 antibody (capture antibody, 1:8,000) in PBS, washed three times (0.05% Tween‐20, PBS) using a Biotek 405US Microplate washer, and blocked for 1 h at room temperature (0.05% Tween‐20, 1% BSA in PBS). Plates were incubated with 80 μl/well of CSF samples diluted with one volume of RIPA buffer (137 mM NaCl, 20 mM Tris pH = 7.5, 10% glycerol, 1% Triton X‐100, 0.5% sodium deoxycholate, 0.1% SDS, 2 mM EDTA) and supplemented with a protease inhibitor cocktail (Sigma) for 2 h at room temperature on a shaking platform. Pseudonymized samples were randomly distributed on the plate and measured blindly in two replicates. After three washing steps, the plates were incubated with MSD sulfo‐tag‐labeled 3F9 antibody (detection antibody, 1:1,000) for 2 h at room temperature on a shaking platform followed by three final washing steps. Upon adding 100 μl MSD Read Buffer T, the plates were immediately measured. The electrochemical signal was detected using a MSD SECTOR Imager 2400. 15‐mer GST‐DPR fusion proteins were purified from *Escherichia coli* as described (Mori *et al*, 2013b). After background correction, data are presented in arbitrary units.

### Measurement of neurofilament levels

Neurofilament, that is, NfL and pNfH, levels were measured using commercial ELISA kits from Quanterix, Lexington (NfL), and BioVendor (pNfH) (Steinacker *et al*, 2016a,b). Values of pNfH below the detection limit (188 pg/ml) were set to 188 pg/ml to permit statistical analysis.

### Statistics

Statistical analysis was performed using GraphPad Prism 5.0 and JMP software 11.1.1. The data did not follow a normal distribution, and therefore, non‐parametric tests were used. Groups were compared by Kruskal–Wallis test and Dunn's *post hoc* test (> 2 groups) or Mann–Whitney test. Correlation analysis was performed with Spearman's rank correlation coefficient. ROC curve analysis was used to calculate sensitivity and specificity of poly‐GP expression, and a threshold to separate *C9orf72* mutation carriers and non‐carriers was selected using the Youden index. A *P*‐value < 0.05 was regarded as statistically significant.

## Author contributions

DE and MO were responsible for conception and design of the study. All authors participated in acquisition and analysis of data. CL, PO, MO, and DE drafted the manuscript and figures.

## Conflict of interest

The authors declare that they have no conflict of interest.

**The paper explained**

**Problem**

A massive expansion of a GGGGCC repeat upstream of the *C9orf72* coding region is the most common genetic cause of ALS and behavioral variant FTD. The expanded repeat is translated in all reading frames into five aggregating dipeptide repeat (DPR) proteins poly‐GA, poly‐GP, poly‐GR, poly‐PA, and poly‐PR. Reliable detection of these proteins *in vivo* would be a desirable clinical biomarker for diagnosis and therapeutic studies. Several DPRs are clearly toxic in cellular and animal models, but their role in human pathogenesis remains controversial. Therefore, we asked how the levels of DPRs differ between asymptomatic carriers and patients with manifest ALS and bvFTD.

**Results**

We developed an immunoassay for poly‐GP in the CSF using two monoclonal antibodies and measured poly‐GP in 125 samples from nine groups, including Parkinson and Alzheimer cases. Screening by immunoassay revealed one misdiagnosed *C9orf72* carrier among the AD cohort. The poly‐GP levels in asymptomatic carriers and ALS/FTD patients are similar, suggesting widespread early expression of DPR proteins consistent with rare autopsy reports. Poly‐GP levels show no correlation with clinical disease stage or other established markers for axonal loss in CSF.

**Impact**

The poly‐GP immunoassay is a useful biomarker for *C9orf72* ALS/FTD cases. DPR expression in the presymptomatic stage may explain the early prodromal brain volume loss and behavior alterations previously observed in *C9orf72* mutation carriers.

## Supporting information



Expanded View Figures PDF

Review Process File
