# Supplementary material for: Poly‐GP in cerebrospinal fluid links C9orf72‐associated dipeptide repeat expression to the asymptomatic phase of ALS/FTD
Source: EMBO Mol Med. 2017 Apr 13;9(7):859–68. doi: 10.15252/emmm.201607486 (PMC5494528; doi:10.15252/emmm.201607486)
Supplement: Supplementary file 1 — Expanded View Figures PDF [file EMMM-9-859-s001.pdf]

Expanded View Figures

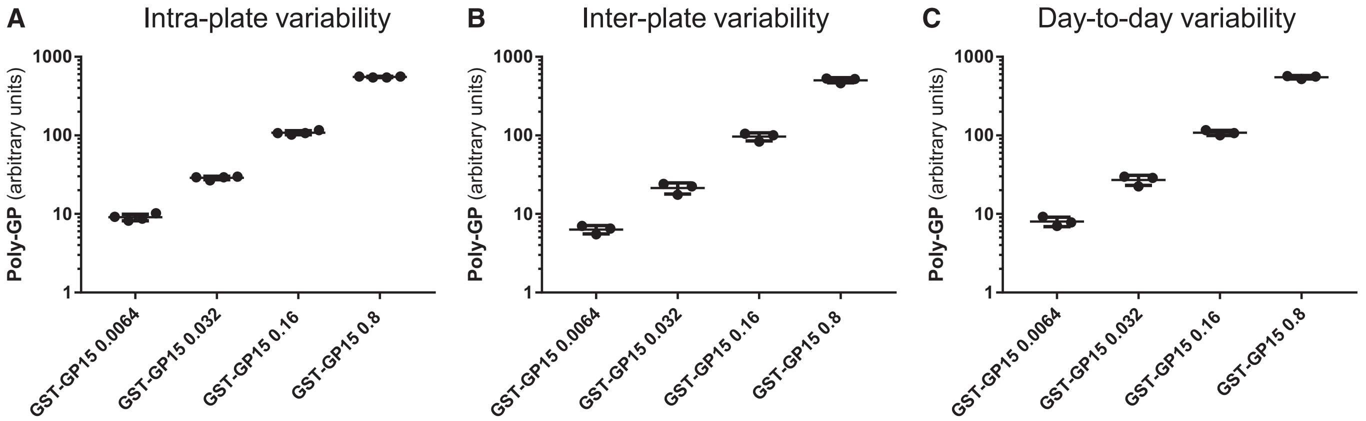

D

	GST-GP15			
	**0.0064 ng/ml**	**0.032 ng/ml**	**0.16 ng/ml**	**0.8 ng/ml**
**Intra-plate variability**				
Mean (AU)	9.08	28.70	108.08	553.83
SD	0.85	1.35	6.13	8.81
CV (%)	9.41	4.72	5.67	1.59
**Inter-plate variability**				
Mean (AU)	6.33	21.33	96.33	503.80
SD	0.76	3.40	11.34	37.07
CV (%)	12.06	15.95	11.77	7.36
**Day-to-day variability**				
Mean (AU)	8.00	27.00	107.83	549.50
SD	1.11	3.92	7.96	26.19
CV (%)	13.92	14.53	7.38	4.77

Figure EV1. The poly-GP immunoassay is reproducible.

A–D Poly-GP sandwich immunoassay with anti-GP antibodies 18H8 and 3F9 was used to analyze the GST-GP_{15} standard at four concentrations. Background-corrected absolute values, mean, and standard deviation (SD) for n = 4 GST-GP_{15} intra-plate replicates (A), n = 3 inter-plate replicates (B), and n = 3 day-to-day replicates (C). Mean, SD, and the coefficient of variance (CV) for all conditions are listed in (D).

© 2017 The Authors
